# LINC00892 as a Prognostic Biomarker in Lung Adenocarcinoma: Role in Immune Infiltration and EMT Suppression

**DOI:** 10.1155/jimr/4341348

**Published:** 2025-04-22

**Authors:** Xinyu Luan, Xuxing Peng, Qinghua Hou, Jixian Liu

**Affiliations:** Department of Thoracic Surgery, Peking University Shenzhen Hospital, Shenzhen, Guangdong, China

**Keywords:** biomarker, immune cells, lung adenocarcinoma, noncoding RNA, prognosis

## Abstract

Lung adenocarcinoma (LUAD) is a prevalent and aggressive form of lung cancer with poor prognosis, largely due to late-stage diagnosis and limited therapeutic options. Recent studies suggest that long noncoding RNAs (lncRNAs) play critical roles in cancer progression and immune modulation, emerging as potential therapeutic targets. In this study, we investigated the expression and functional role of LINC00892 in LUAD using RNA sequencing data from The Cancer Genome Atlas (TCGA) and functional assays in vitro and in vivo. We found that LINC00892 is significantly downregulated in LUAD tissues compared to normal tissues, and lower LINC00892 expression correlates with poorer overall survival (OS), disease-specific survival (DSS), and progression-free interval (PFI), particularly in younger patients and those with early-stage disease. Bioinformatic analyses revealed that LINC00892 expression is positively correlated with immune cell infiltration, including CD4^+^ and CD8^+^ T cells, and negatively correlated with tumor-promoting Th2 cells, suggesting its role in shaping the tumor immune microenvironment. In vitro functional assays showed that LINC00892 overexpression inhibits LUAD cell proliferation, migration, and invasion while promoting apoptosis. Mechanistically, LINC00892 upregulation was found to suppress epithelial–mesenchymal transition (EMT) by increasing E-cadherin expression and decreasing levels of N-cadherin, vimentin, and slug. Additionally, in an in vivo mouse xenograft model, LINC00892 overexpression suppressed tumor growth and metastasis, accompanied by enhanced immune cell infiltration such as CD4^+^ and CD8^+^ T cells. Collectively, these findings suggest that LINC00892 acts as a tumor suppressor in LUAD by modulating immune infiltration and EMT, highlighting its potential as a prognostic biomarker and therapeutic target.

## 1. Introduction

Lung cancer remains one of the most lethal forms of malignancies worldwide, with lung adenocarcinoma (LUAD) accounting for ~50% of all lung cancer cases [1, 2]. Despite significant advancements in treatment strategies, including surgery, chemotherapy, radiotherapy, and immunotherapy, the overall survival (OS) rate for LUAD patients has not shown notable improvement [3]. A significant challenge lies in the lack of effective early screening tools and the often asymptomatic nature of early-stage LUAD, leading to late diagnoses when the disease has already advanced or metastasized [4]. This highlights the critical need for a better understanding of LUAD's molecular mechanisms to identify novel therapeutic targets.

Recent efforts have focused on dissecting the molecular drivers of LUAD through large-scale studies like whole genome sequencing, RNA sequencing, and proteomics [5]. However, the underlying pathogenesis of LUAD remains poorly understood, which continues to limit the development of more effective treatments. As such, the identification of new molecular targets is essential to improve clinical outcomes and provide precision-based therapies for LUAD patients.

Long noncoding RNAs (lncRNAs), which are RNA molecules longer than 200 nucleotides without protein-coding potential, have emerged as significant regulators in various biological processes [6]. These processes include cell differentiation, growth, apoptosis, epithelial–mesenchymal transition (EMT), and immune responses [7]. LncRNAs exert their functions at multiple regulatory levels—epigenetic, transcriptional, and post-transcriptional [8]. Importantly, numerous studies have shown that dysregulated expression of lncRNAs plays a pivotal role in cancer development, including tumorigenesis, metastasis, and angiogenesis [9]. Given this, lncRNAs are increasingly viewed as potential therapeutic targets and prognostic biomarkers [10]. Several studies have highlighted specific lncRNAs as key players in cancer. For example, Jing et al. [11] demonstrated that lncRNA LL22NC03-N64E9.1 promotes the proliferation of lung cancer cells and serves as a potential prognostic marker. Similarly, Lan et al. identified lncRNA XIST as a biomarker for predicting breast cancer recurrence [12], while Yan et al. [13] revealed the diagnostic relevance of lncRNA PCAT14 in immune cell infiltration and prostate cancer prognosis. These findings underline the potential of lncRNAs as biomarkers across different cancer types.

Among the novel lncRNAs, LINC00892 has garnered attention for its role in the immune microenvironment. It was initially reported to be induced in T cells and expressed in follicular lymphoma-resident T-helper cells [14]. Research by Chen et al. [15] suggests that LINC00892 may have prognostic value in bladder cancer, indicating its potential as a therapeutic target. Another study has predicted that LINC00892 is a biomarker associated with activated T cells in LUAD [16]. Additionally, a predictive model identified LINC00892 as a diagnostic biomarker for detecting N2 metastasis in LUAD [17]. Moreover, LINC00892 was identified as one of the ferroptosis-related lncRNAs involved in the prognosis of non–small cell lung cancer (NSCLC), contributing to the development of a predictive model for patient outcomes [18]. Despite these insights, the functional role of LINC00892 in LUAD, particularly concerning its influence on the tumor microenvironment (TME) and immune infiltration, remains unclear.

Given the critical role of immune infiltration in cancer progression and prognosis [19], understanding the contribution of LINC00892 to these processes is of paramount importance. Therefore, our study aims to investigate the expression of LINC00892 in LUAD tissues using RNA-seq data from The Cancer Genome Atlas (TCGA). We sought to assess its prognostic value and explore its relationship with immune infiltration using single-sample gene set enrichment analysis (ssGSEA). Additionally, we performed functional assays to examine the biofunctional role of LINC00892 in LUAD. Understanding this association may not only uncover new avenues for therapeutic interventions but also improve the ability to predict patient outcomes, providing a rationale for the urgent need to conduct this study.

## 2. Materials and Methods

### 2.1. Bioinformatics Analysis

Gene expression data, along with clinical information for LUAD patients (572 cases, HTSeq-FPKM workflow), were retrieved from the TCGA database (https://portal.gdc.cancer.gov/). The level 3 HTSeq-FPKM data were normalized to transcripts per million (TPM) for subsequent analyses. Differentially expressed genes (DEGs) between high and low LINC00892 expression groups were identified using the “DESeq2” package [20], with thresholds set at |log_2_FC| > 1.5 and an adjusted *p*-value  < 0.05.

### 2.2. ssGSEA

Immune cell infiltration in LUAD was quantified using the ssGSEA method from the R package GSVA [21]. Infiltration abundances of 24 immune cell types were calculated based on 509 gene signatures [22]. To examine the correlation between LINC00892 expression and immune cell infiltration, statistical significance was assessed using Spearman correlation and the Wilcoxon rank sum test.

### 2.3. Clinical Samples

Tumor and adjacent nontumor tissue samples were collected from 30 LUAD patients who underwent surgical resection at Peking University Shenzhen Hospital between March 2020 and September 2022. All samples were immediately preserved in liquid nitrogen following excision. This study was approved by the Ethics Committee of Peking University Shenzhen Hospital, and written informed consent was obtained from all patients. None of the patients had received chemotherapy or radiotherapy prior to surgery.

### 2.4. Cell Culture

Normal human bronchial epithelial (HBE) cells were purchased from Procell (Wuhan, China), while LUAD cell lines NCI-H23, A549, and HCC827 were purchased from American Type Culture Collection (CA, USA). All these cell lines were cultured in DMEM supplemented with 10% fetal bovine serum (FBS) (Gibco, Carlsbad, CA) and incubated at 37°C in a humidified atmosphere with 5% CO_2_.

### 2.5. Cell Transfection

To overexpress LINC00892 in NCI-H23 and A549 cells, the full-length sequence of LINC00892 was cloned into the pcDNA3.1 expression vector (Geenseed Biotech, Guangzhou, China). An empty pcDNA3.1 vector was used as a negative control. Cell transfection was carried out using Lipofectamine 3000 reagent (Invitrogen, USA) according to the manufacturer's instructions, and the cells were incubated for 48 h following transfection to ensure sufficient expression of LINC00892.

### 2.6. RT-qPCR

Total RNA was isolated from cells using the TRIzol reagent (Invitrogen, USA) following the manufacturer's instructions. The concentration and purity of the RNA were assessed using a NanoDrop spectrophotometer (Thermo Scientific, USA). For reverse transcription, 1 μg of total RNA was used to synthesize cDNA with the PrimeScript RT reagent kit (Takara, Dalian, China), following the kit protocol. Quantitative real-time PCR (qPCR) was performed using the SYBR Green detection method (Takara) on a CFX96 real-time PCR system (Bio-Rad, USA). Target gene expression was normalized to a reference gene, and relative gene expression was calculated using the 2^−ΔΔCt^ method. The primer sequences used in this study are LINC00892 forward, 5′-TGGATGTTCTTTGCTGGGCT-3′, reverse, 5′-GCTCGTTCTTCTCTTACGGCT-3′, and GAPDH forward, 5′-GTCAAGGCTGAGAACGGGAA-3′, reverse, 5′-AAATGAGCCCCAGCCTTCTC-3′.

### 2.7. Western Blot

Total proteins were extracted from cells and tissues using RIPA buffer with protease and phosphatase inhibitors. Protein concentrations were measured using the BCA assay. Equal amounts of protein were separated on a 12% SDS-PAGE gel and transferred onto PVDF membranes (Millipore, USA). The membranes were blocked with 5% nonfat milk in TBST for 1 h and then incubated overnight at 4°C with primary antibodies (Abcam, USA). After washing with TBST, membranes were incubated with HRP-conjugated secondary antibodies (Abcam, USA) for 2 h. Proteins were visualized using an enhanced chemiluminescence (ECL) detection system, and protein expression was quantified using ImageJ software.

### 2.8. Cell Counting Kit-8 (CCK-8) Assay

The CCK-8 (Dojindo, Japan) was used to assess cell viability. NCI-H23 and A549 cells were seeded in 96-well plates at a density of 1 × 10^4^ cells per well and allowed to adhere for 24 h. After 24, 48, and 72 h of incubation, 10 μL of CCK-8 reagent was added to each well. The plates were then incubated for an additional 2 h at 37 °C. The absorbance was measured at 450 nm using a microplate reader (Molecular Devices, USA) to quantify cell viability.

### 2.9. Colony Formation Assay

Cell proliferative capability was assessed by colony formation assay. In brief, NCI-H23 and A549 cells were seeded into 6-well plates at a density of 1000 cells per well and incubated at 37°C with 5% CO_2_ for approximately 2 weeks to allow colony formation. The medium was replaced every 3–4 days. After the incubation period, the colonies were carefully washed with phosphate-buffered saline (PBS), fixed with 4% paraformaldehyde for 20 min at room temperature, and stained with 0.1% crystal violet solution for 30 min. Excess dye was removed by rinsing the plates with PBS. The stained colonies were visualized and counted under a microscope (Olympus, Tokyo, Japan).

### 2.10. EdU Staining

The EdU incorporation assay was performed using the EdU Assay Kit (RiboBio, China) following the manufacturer's protocol. Briefly, NCI-H23 and A549 cells were seeded into 96-well plates and incubated for 24 h at 37 °C in a humidified atmosphere with 5% CO_2_. After incubation, 100 μL of fresh medium containing 50 μM EdU was added to each well, and the cells were incubated for an additional 2 h to allow EdU incorporation into newly synthesized DNA. Following the incubation, the cells were fixed with 4% paraformaldehyde for 15 min at room temperature and permeabilized using 0.5% Triton X-100 for 10 min. Next, the cells were treated with the Apollo reaction cocktail according to the kit instructions to fluorescently label the incorporated EdU. Subsequently, the cells were counterstained with Hoechst dye to visualize the nuclei. The stained cells were then observed and imaged using a fluorescence microscope (Olympus, Japan). The number of EdU-positive cells was quantified, and the percentage of proliferating cells was calculated.

### 2.11. Flow Cytometry

The cell apoptosis rate was evaluated using the Annexin V-FITC Apoptosis Detection Kit (BD Biosciences) following the manufacturer's instructions. Briefly, NCI-H23 and A549 cells were harvested after the respective treatments and washed twice with cold PBS. The cells were then resuspended in 1× binding buffer at a concentration of 1 × 10^6^ cells/mL. Next, 5 μL of Annexin V-FITC and 5 μL of propidium iodide (PI) were added to each sample, and the cells were incubated in the dark at room temperature for 15 min. Following incubation, the stained cells were acquired using a flow cytometer (FACS Calibur, BD Biosciences). Finally, data were analyzed using FlowJo software, and the percentage of apoptotic cells was quantified.

### 2.12. Transwell Assay

The Transwell assay was used to assess cell migration and invasion. For the migration assay, NCI-H23 and A549 cells in 200 μL of serum-free medium were seeded in the upper chamber of a Transwell insert with 8.0 μm pores (Corning, USA). For the invasion assay, the upper chambers were precoated with Matrigel. The lower chambers were filled with 600 μL of complete medium containing 10% FBS. After 24 h of incubation at 37°C, nonmigrated or noninvaded cells were removed from the upper surface, while the migrated or invaded cells on the lower surface were fixed with 4% paraformaldehyde and stained with 0.1% crystal violet. The cells were counted under a light microscope (Olympus, Japan), and results were averaged from five random fields.

### 2.13. Wound Healing Assay

The wound healing assay was performed to evaluate cell migration. NCI-H23 and A549 cells were seeded into 6-well plates and cultured until they reached ~90% confluence. A 200 μL pipette tip was used to create a straight scratch across the cell monolayer. The detached cells were gently washed away with PBS to ensure a clean wound area. The cells were then cultured in serum-free EBM-2 medium to prevent cell proliferation and focus on migration. Images of the wound area were captured using a microscope (Olympus, Japan) immediately after scratching (0 h) and again after 24 h of incubation at 37°C with 5% CO_2_. The wound closure was assessed by comparing the images at the two time points, and the relative migration rate was calculated.

### 2.14. Mouse Xenograft Tumor Model

Ten female BALB/c nude mice, aged between 4 and 6 weeks and weighing 18 ± 5 g, were obtained from Vital River Laboratories in Beijing, China. The mice were housed under standard laboratory conditions. All experimental protocols were reviewed and approved by the Experimental Animal Ethics Committee of Peking University Shenzhen Hospital. The mice were randomly assigned into two groups: one group received pcDNA3.1-NC, and the other group received pcDNA3.1-LINC00892, with five mice in each group. To establish xenograft models, 5 × 10^6^ A549 cells transfected with either pcDNA3.1-NC or pcDNA3.1-LINC00892 were subcutaneously injected into the dorsal area of each mouse. Tumor size was recorded at 5-day intervals. After 30 days, the mice were sacrificed, and the tumors were removed, photographed, and weighed for further analysis.

To assess metastasis, tumor samples were fixed in 4% paraformaldehyde and sectioned into 4 μm slices. Hematoxylin and eosin (H&E) staining was performed on the tumor sections to examine the histopathology. The stained sections were analyzed using a light microscope (Olympus, Japan) to determine the presence and extent of metastasis.

### 2.15. Immunohistochemistry (IHC)

IHC was performed on paraffin-embedded tumor tissues. The tissues were sectioned into 4-μm-thick slices and subsequently dewaxed in xylene and rehydrated through a graded ethanol series. Antigen retrieval was carried out by heating the sections in citrate buffer (pH 6.0) for 10–15 min. After cooling to room temperature, the sections were blocked with 3% hydrogen peroxide to inhibit endogenous peroxidase activity. The tissue sections were then incubated overnight at 4°C with primary antibodies (Abcam, USA) diluted in PBS. Following incubation, the sections were washed with PBS and incubated with HRP-conjugated secondary antibodies (Abcam, USA) for 2 h at room temperature. The signal was developed using 3,3′-diaminobenzidine (DAB) as the chromogenic substrate, producing a brown color at the site of antibody binding. Finally, the sections were counterstained with hematoxylin, dehydrated, and mounted. The stained sections were examined and photographed under a light microscope (Olympus, Japan) to assess protein expression levels. The IHC results were quantified by analyzing staining intensity and the percentage of positive cells in five random fields for each sample.

### 2.16. Statistical Analysis

Statistical analyses were performed using R software (v.3.6.3) and GraphPad Prism (v.7.0). To assess the prognostic value of LINC00892 in LUAD, Cox proportional hazards regression analysis was conducted for OS, disease-specific survival (DSS), and progression-free interval (PFI). Clinical and pathological subgroup analyses were performed using the “forestplot” R package to visualize the prognostic significance in different patient cohorts. All experiments were conducted with biological replicates, with each experiment repeated in triplicate. Data are presented as the mean ± standard deviation (SD). Group comparisons were made using a two-tailed Student's *t*-test, and *p*-values less than 0.05 were considered statistically significant

## 3. Results

### 3.1. LINC00892 Is Downregulated in LUAD

**Figure 1:**
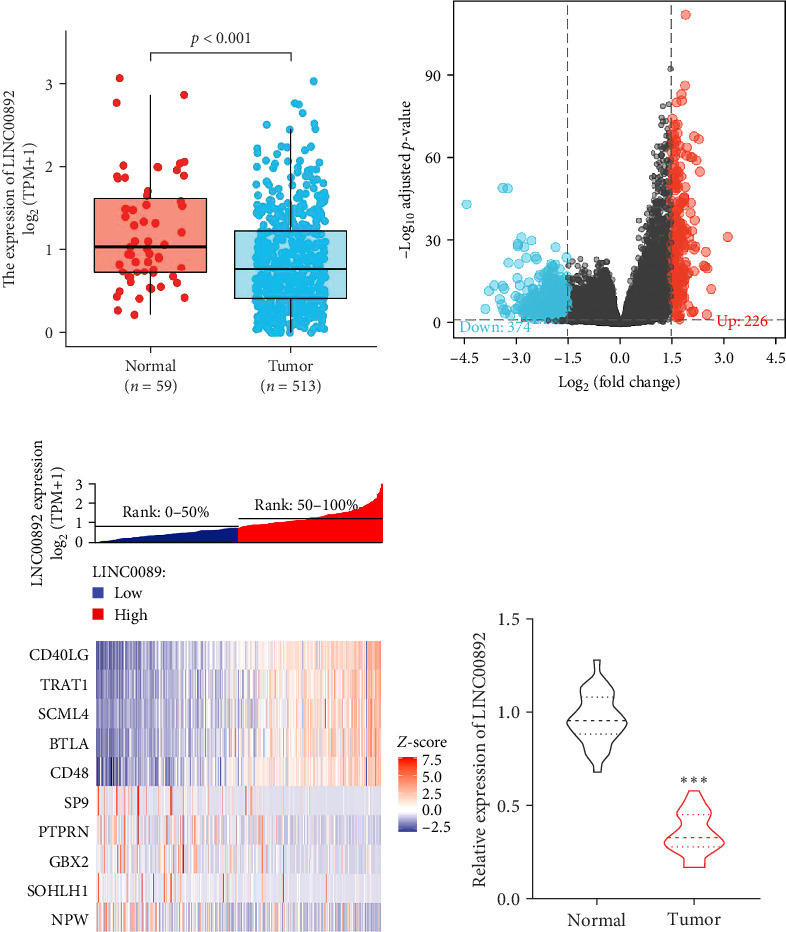
LINC00892 is downregulated in LUAD. (A) The Cancer Genome Atlas (TCGA) database was accessed to explore LINC00892 expression levels in LUAD tissues (*n* = 513) and adjacent normal tissues (*n* = 59). (B) Volcano plot illustrating differentially expressed genes (DEGs). (C) Relative expression levels of the top 10 DEGs between the high and low LINC00892 expression groups. (D) RT-qPCR was performed to measure LINC00892 expression in LUAD tissues (*n* = 30) and normal adjacent tissues (*n* = 30). *⁣*^*∗∗∗*^*p* < 0.001.

To investigate the expression of LINC00892 in LUAD, we assessed its expression levels in 513 LUAD tissues and 59 normal lung tissues using the TCGA database. Compared to normal tissues, LINC00892 expression was significantly lower in LUAD tumors (Figure 1A). Through differential expression analysis using the “DESeq2” package on the TCGA dataset, we identified 600 DEGs, including 226 upregulated and 374 downregulated genes (Figure 1B). The relative expression levels of the top 10 DEGs in both high and low LINC00892 expression groups were visualized (Figure 1C). Consistent with these findings, RT-qPCR analysis demonstrated that LINC00892 was expressed at lower levels in LUAD tissues compared to their paired normal counterparts (Figure 1D). This data suggest that LINC00892 expression is low in LAUD.

### 3.2. LINC00892 Expression Is Associated With Immune Infiltration

**Figure 2:**
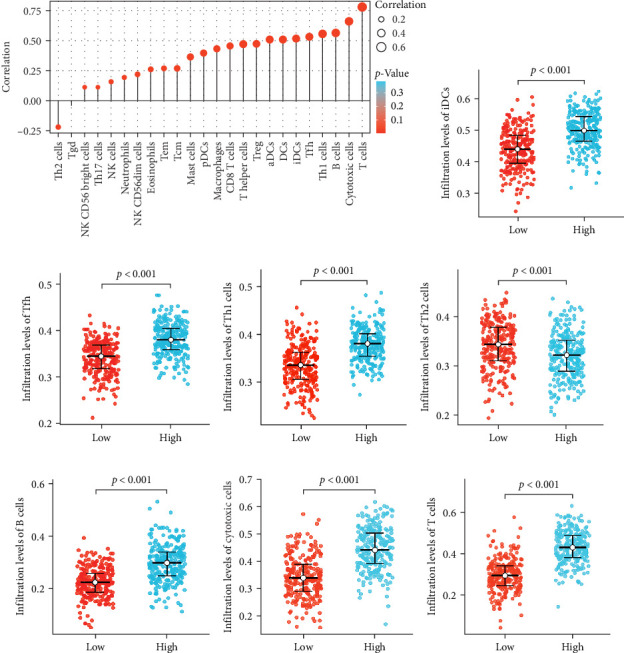
LINC00892 expression is associated with immune cell infiltration. (A) Correlation analysis between LINC00892 expression and the abundance of 24 different immune cell types. (B–H) Comparison of infiltration levels of immature dendritic cells (iDCs), T follicular helper (Tfh) cells, Th1 cells, Th2 cells, B cells, cytotoxic cells, and T cells in tumor tissues between the high LINC00892 expression group and the low LINC00892 expression group. *⁣*^*∗∗∗*^*p* < 0.001.

Given the known role of immune cell infiltration in cancer progression, we next examined whether LINC00892 expression was correlated with immune infiltration in LUAD. We used Spearman's correlation analysis and ssGSEA to examine this association. The results demonstrated that LINC00892 expression positively correlated with several immune cell types, including immature dendritic cells (iDCs), T follicular helper (Tfh) cells, T-helper 1 (Th1) cells, B cells, cytotoxic cells, and T cells while showing a negative correlation with Th2 cells (Figure 2). These results suggest that LINC00892 may influence the tumor immune microenvironment in LUAD, which could contribute to its impact on tumor progression.

### 3.3. Prognostic Value of LINC00892 in the LUAD Clinicopathological Subgroups

**Figure 3:**
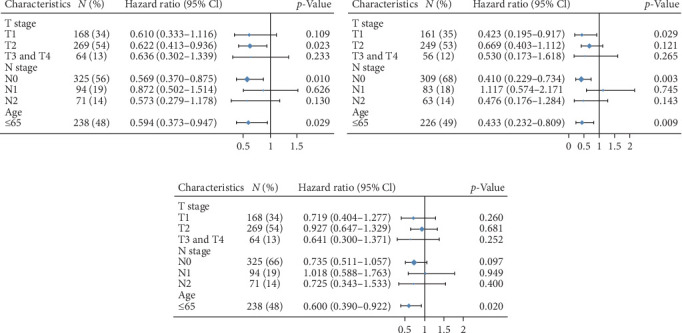
Prognostic performance of LINC00892 in the LUAD clinicopathological subgroups. The prognostic value of LINC00892 expression was evaluated for clinical outcomes in various LUAD patient subgroups. Cox proportional hazards analysis was used to assess the impact of LINC00892 expression on (A) overall survival (OS), (B) disease-specific survival (DSS), and (C) progression-free interval (PFI).

To further investigate the prognostic significance of LINC00892 in LUAD, we performed a subgroup analysis of clinicopathological factors using Cox proportional hazards models, with results displayed as forest plots. We found that lower LINC00892 expression was associated with poorer OS in patients with T2 stage (HR = 0.622, 95% CI: 0.413–0.936, *p*=0.023), N0 stage (HR = 0.569, 95% CI: 0.370–0.875, *p*=0.010), and in patients younger than 65 years (HR = 0.594, 95% CI: 0.373–0.947, *p*=0.029) (Figure 3A). LINC00892 was also a predictor of DSS in patients with T1 stage (HR = 0.423, 95% CI: 0.195–0.917, *p*=0.029), N0 stage (HR = 0.410, 95% CI: 0.229–0.734, *p*=0.003), and in those younger than 65 years (HR = 0.433, 95% CI: 0.232–0.809, *p*=0.009) (Figure 3B). Furthermore, lower LINC00892 expression was associated with reduced PFI in patients with N0 stage (HR = 0.735, 95% CI: 0.511–1.057, *p*=0.097) and in those under 65 years of age (HR = 0.600, 95% CI: 0.390–0.922, *p*=0.020) (Figure 3C). Thus, LINC00892 appears to serve as a prognostic marker in LUAD. These results collectively suggest that LINC00892 may serve as a prognostic biomarker in LUAD patients, particularly in younger individuals and those with less advanced disease stages.

### 3.4. LINC00892 Upregulation Inhibits Cell Proliferation and Promotes Apoptosis in LUAD

**Figure 4:**
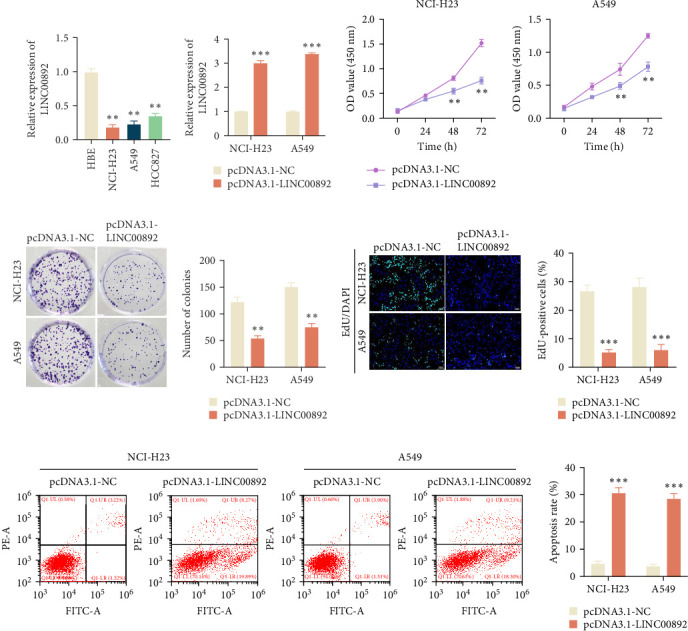
LINC00892 upregulation inhibits LUAD cell growth by promoting apoptosis and reducing proliferation. (A) RT-qPCR analysis of LINC00892 expression in LUAD cell lines (NCI-H23, A549, and HCC827) compared to the normal human bronchial epithelial (HBE) cell line. (B) Transfection efficiency of pcDNA3.1-LINC00892 in NCI-H23 and A549 cells, as determined by RT-qPCR. (C) Cell viability was assessed using the CCK-8 assay in the pcDNA3.1-LINC00892 and pcDNA3.1-NC groups. (D, E) Cell proliferation was evaluated by colony formation and EdU assays in indicated groups. (F) Cell apoptosis was measured via flow cytometry. Data are shown as the mean ± SD of four independent experiments, and statistical analyses were performed using a two-tailed Student's *t*-test. *⁣*^*∗∗*^*p* < 0.01, *⁣*^*∗∗∗*^*p* < 0.001.

In the next step, we evaluated the biological function of LINC00892 in LUAD progression. To this end, we first assessed the expression of LINC00892 in normal HBE cells and various LUAD cell lines, including NCI-H23, A549, and HCC827. Results showed that LINC00892 was significantly downregulated in LAUD cell lines compared to the control HBE cells (Figure 4A). We then performed functional assays following LINC00892 overexpression in LUAD cell lines. The overexpression of LINC00892 was achieved via transfection with pcDNA3.1-LINC00892, as verified by RT-qPCR (Figure 4B). Cell viability assays (CCK-8) revealed that LINC00892 overexpression significantly reduced the viability of LUAD cells (Figure 4C). Similarly, colony formation assays showed a marked reduction in colony formation upon LINC00892 overexpression (Figure 4D). EdU assays further confirmed that LINC00892 upregulation significantly decreased the number of EdU-positive cells, indicating that LINC00892 suppresses cell proliferation (Figure 4E). Additionally, flow cytometry analysis revealed that LINC00892 overexpression significantly increased the apoptotic rate in LUAD cells (Figure 4F). Taken together, these results indicate that LINC00892 upregulation inhibits LUAD cell growth by promoting apoptosis and reducing proliferation.

### 3.5. LINC00892 Upregulation Suppresses EMT and Metastasis in LUAD

**Figure 5:**
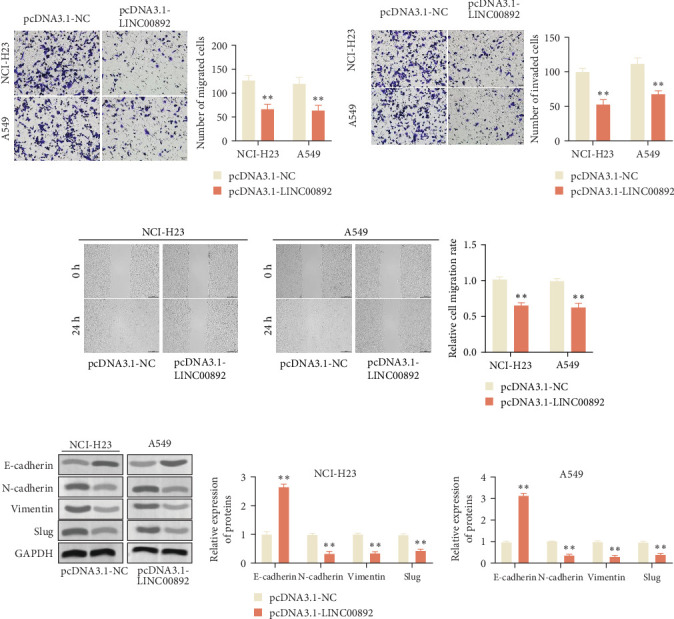
LINC00892 upregulation suppresses epithelial–mesenchymal transition (EMT) and cell migration in LUAD. (A–C) Cell migration and invasion were assessed using Transwell and wound healing assays in the pcDNA3.1-LINC00892 and pcDNA3.1-NC groups. (D) Western blot analysis of EMT markers, including E-cadherin, N-cadherin, vimentin, and slug, in different cell lines. Data are shown as the mean ± SD of four independent experiments, and statistical analyses were performed using a two-tailed Student's *t*-test. *⁣*^*∗∗*^*p* < 0.01.

To further investigate the effects of LINC00892 on tumor progression, we assessed its impact on EMT, migration, and invasion. Transwell assays showed that LINC00892 overexpression significantly reduced both migratory and invasive capabilities of LUAD cells (Figure 5). Consistently, wound healing assays further confirmed that LINC00892 upregulation inhibited cell migration (Figure 5C). Mechanistically, western blot analysis demonstrated that LINC00892 overexpression increased E-cadherin expression while reducing N-cadherin, vimentin, and slug levels, markers associated with EMT (Figure 5D). These findings suggest that LINC00892 upregulation inhibits EMT and suppresses the migratory and invasive potential of LUAD cells.

### 3.6. LINC00892 Upregulation Inhibits Tumor Growth and Metastasis In Vivo

**Figure 6:**
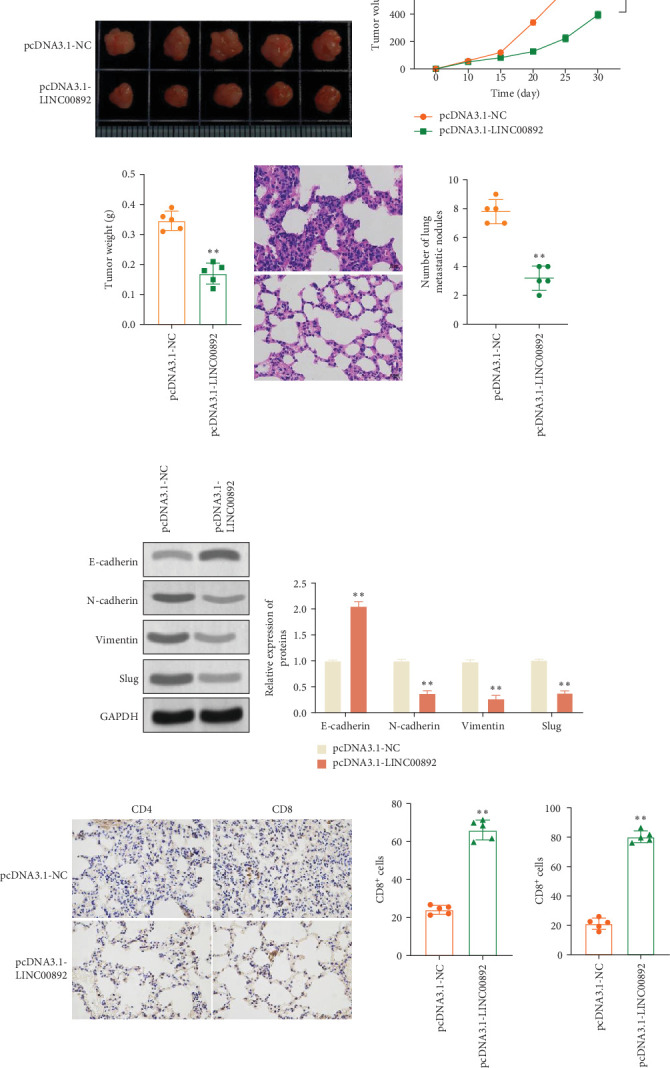
LINC00892 upregulation suppresses tumor growth and metastasis in vivo. (A) Representative images of tumors from the pcDNA3.1-LINC00892 group and the pcDNA3.1-NC group. (B, C) Tumor volume and weight were measured in mice from both the pcDNA3.1-LINC00892 and pcDNA3.1-NC groups. (D) H&E staining of lung sections showing metastatic nodules. A scatter plot was used to quantify the number of lung metastases (*n* = 5). (E) Western blot analysis of EMT-related proteins, including E-cadherin, N-cadherin, vimentin, and slug, in tumor tissues. (F) Immunohistochemical (IHC) staining was used to assess the number of CD4^+^ and CD8^+^ T cells in tumor tissues. Data are shown as the mean ± SD from five mice per group, and statistical analyses were performed using a two-tailed Student's *t*-test. *⁣*^*∗∗*^*p* < 0.01, *⁣*^*∗∗∗*^*p* < 0.001.

Finally, we investigated the in vivo effects of LINC00892 on tumor growth by establishing a xenograft model in mice by injecting A549 cells stably transfected with either pcDNA3.1-NC or pcDNA3.1-LINC00892. Tumors from mice injected with LINC00892-overexpressing cells showed significantly reduced growth, volume, and weight compared to the control group (Figure 6). Histopathological evaluation (H&E staining) revealed a notable decrease in the number of metastatic nodules in the LINC00892-overexpressing group compared to the control group (Figure 6D). Western blot analysis further demonstrated elevated E-cadherin expression and reduced levels of N-cadherin, vimentin, and slug in tumor tissues from the LINC00892-overexpressing group compared to the control group (Figure 6E). Moreover, IHC results indicated that LINC00892 upregulation enhanced CD4^+^ and CD8^+^ T cell infiltration into the TME (Figure 6F). Collectively, these findings suggest that LINC00892 suppresses tumor growth and metastasis by promoting immune cell infiltration and modulating EMT in LUAD.

## 4. Discussion

LncRNAs are increasingly recognized for their crucial roles in regulating gene expression and contributing to cancer progression through various mechanisms [23]. Emerging evidence suggests that dysregulated expression of lncRNAs is involved in numerous biological processes in cancers, including LUAD [24–26]. Several studies have identified aberrantly expressed lncRNAs in LUAD, demonstrating their oncogenic or tumor-suppressive functions [27, 28].

In our study, we identified LINC00892 as a novel lncRNA that is significantly downregulated in LUAD tissues, as confirmed by both the TCGA bioinformatics analysis and RT-qPCR validation. These findings suggest that LINC00892 may serve as a potential biomarker for LUAD. Our results align with prior research on LINC00892 in other cancers. For instance, LINC00892 has been reported to be a predictor of prognosis and immunotherapeutic response in bladder cancer [29]. Furthermore, Hao et al. [17] proposed a diagnostic model incorporating LINC00892 as a biomarker for metastasis in LUAD. This consistent evidence across different cancer types supports our hypothesis that LINC00892 plays a pivotal role in LUAD progression. In this context, our study adds to the growing body of evidence highlighting the prognostic value of LINC00892, as our Cox analysis demonstrated that lower LINC00892 expression is associated with poorer OS, DSS, and PFI in LUAD patients, especially those with less advanced disease stages and younger patients.

Additionally, the TME plays a critical role in cancer development and progression [30]. The TME comprises various components, including tumor cells, stromal cells, immune cells, and secreted cytokines [31]. Tumor-infiltrating immune cells and their associated factors are key elements of the TME and are often linked to clinical outcomes in cancer patients [32]. Studies have demonstrated that immune cell infiltration in tumors is closely related to clinical outcomes in patients [33]. In our study, we used ssGSEA and Spearman's correlation analysis to investigate the relationship between LINC00892 expression and immune cell infiltration in LUAD. Our results showed that LINC00892 expression was positively correlated with immune cell populations, such as iDCs, Tfh cells, Th1 cells, B cells, cytotoxic cells, and T cells. Notably, there was a negative correlation with Th2 cells, which are typically associated with tumor-promoting immune responses [34]. These results are consistent with previous studies demonstrating the role of T cells, particularly CD8^+^ and CD4^+^ T cells, in exerting antitumor immune responses [35–37]. B cells, through the production of IFNγ and IL-12p40, also contribute to the recruitment and activation of CD8+ T cells, further enhancing tumor control [38]. Therefore, we suggest that LINC00892 may modulate the immune microenvironment in LUAD by influencing the infiltration of immune cells, which could have important implications for immunotherapy.

Furthermore, we investigated the biological function of LINC00892 in LUAD progression. Functional assays revealed that LINC00892 overexpression significantly suppressed LUAD cell proliferation, migration, and invasion while promoting apoptosis. These findings are consistent with the established role of lncRNAs in regulating tumor cell growth and metastasis [39–41]. Our study also demonstrated that LINC00892 overexpression inhibited the EMT process by increasing E-cadherin expression and decreasing the expression of EMT-related markers such as N-cadherin, vimentin, and slug. The inhibition of EMT by LINC00892 aligns with previous research showing that EMT is critical for cancer metastasis and drug resistance [42–45].

In vivo, LINC00892 overexpression suppressed tumor growth and metastasis in a xenograft mouse model, further supporting its tumor-suppressive function. Additionally, LINC00892 overexpression enhanced the infiltration of CD4^+^ and CD8^+^ T cells into the TME, suggesting a potential mechanism by which LINC00892 modulates immune responses in LUAD. These findings are consistent with previous studies that highlight the critical role of immune cell infiltration, particularly T cells, in shaping tumor behavior and improving outcomes in cancer therapy. For instance, LINC00460 is reported to play an important role in pancreatic cancer by regulating immune cell infiltration [46]. Similarly, another study reported that lncRNA NEAT1 interacts with DNMT1 to regulate cytotoxic T cell infiltration by inhibiting cGAS/STING pathway in lung cancer [47]. These studies together with our findings suggest that modulating immune cell infiltration can have profound effects on tumor immunity, reinforcing the idea that lncRNAs like LINC00892 may be promising targets for immunotherapy strategies in LUAD.

Despite the promising findings, our study has certain limitations that need to be addressed. First, although we have demonstrated the role of LINC00892 in modulating immune cell infiltration, the precise molecular mechanisms underlying this effect remain unclear and require further investigation. Second, while LINC00892 seems to promote T cell infiltration, its specific interaction with immune checkpoint pathways is not yet fully understood. Future research should explore whether LINC00892 influences PD-1/PD-L1 signaling or other immune regulatory pathways to better clarify its therapeutic potential in LUAD. Lastly, our in vivo experiments were limited to xenograft models. Additional studies using patient-derived xenografts or genetically engineered mouse models are necessary to validate the therapeutic efficacy of LINC00892 in LUAD.

## 5. Conclusions

Taken together, our study identifies LINC00892 as a novel lncRNA that is downregulated in LUAD and serves as a potential biomarker for prognosis and immune modulation. The overexpression of LINC00892 suppresses tumor growth and metastasis by promoting immune cell infiltration and modulating EMT in LUAD. These findings highlight the potential of LINC00892 as a therapeutic target in LUAD.

## Data Availability

The datasets generated during and/or analyzed during the current study are available from the corresponding author upon reasonable request.
